# Endovascular repair of renal artery anastomotic pseudoaneurysm following thoracoabdominal graft replacement

**DOI:** 10.1016/j.jvscit.2025.101864

**Published:** 2025-05-28

**Authors:** Masaro Nakae, Tetsuro Nakazawa, Hiroki Tada, Naosumi Sekiya, Teruya Nakamura

**Affiliations:** a Department of Cardiovascular Surgery, Sakurabashi Watanabe Advanced Healthcare Hospital, Osaka, Osaka, Japan; b Department of Diagnostic Imaging, Osaka General Medical Center, Osaka, Osaka, Japan

**Keywords:** Pseudoaneurysm, Endovascular repair, Stent graft, Less invasive

## Abstract

Anastomotic pseudoaneurysm following thoracoabdominal graft replacement is a rare but potentially life-threatening complication. Although surgical repair remains the definitive treatment, it is often significantly more invasive than the initial procedure owing to extensive tissue adhesion. Patients with severe frailty are generally not considered suitable candidates for such highly invasive interventions. We present a successful case of endovascular repair of a right renal artery anastomotic pseudoaneurysm using a small-diameter stent graft and coils in an elderly patient with high-grade frailty.

Anastomotic pseudoaneurysm is a rare and serious postoperative complication after thoracoabdominal aortic graft replacement, visceral transplantation, and other surgical procedures.^1^^,^^2^ Although open surgical repair is considered the definitive treatment, it is highly invasive and associated with significant morbidity and mortality. For most older patients with frailty, redo open surgery might be an intolerable procedure. In this report, we describe the successful endovascular repair of a renal artery anastomotic pseudoaneurysm after graft replacement for a thoracoabdominal aortic aneurysm. Written informed consent was obtained from the patient for the publication of this case report.

## Case report

**Fig 1:**
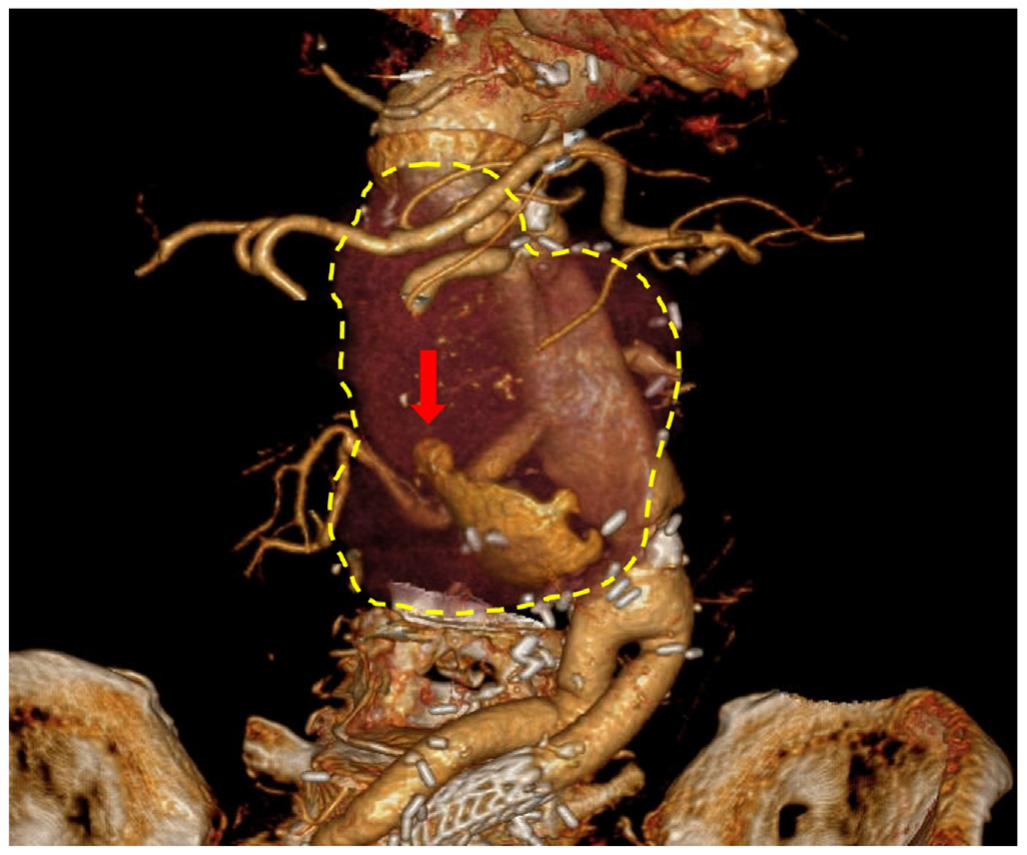
The *yellow dashed line* demonstrates the extent of right renal artery anastomotic pseudoaneurysm. The *red arrow* shows the blood flow into pseudoaneurysm.

A 79-year-old male patient with multiple degenerative aortic aneurysms, who had previously undergone three open surgical repairs for descending, thoracoabdominal, and abdominal aortic aneurysms, as well as endovascular aortic repair for a left internal iliac artery aneurysm, presented to our hospital with acute lower back pain four years after thoracoabdominal aortic graft replacement. Contrast-enhanced computed tomography revealed a significant enlargement of a right renal artery anastomotic pseudoaneurysm, with a maximum diameter of 67 mm (Fig 1). In the absence of elevated inflammatory markers, fever, or positive blood cultures, infection was considered unlikely, and the etiology was attributed to degeneration at the anastomotic site. Although urgent intervention was required, the patient's high degree of frailty rendered open surgical repair unsuitable. Therefore, endovascular repair using small-diameter expanded polytetrafluoroethylene stent grafts was planned to minimize both morbidity and mortality.

**Fig 2:**
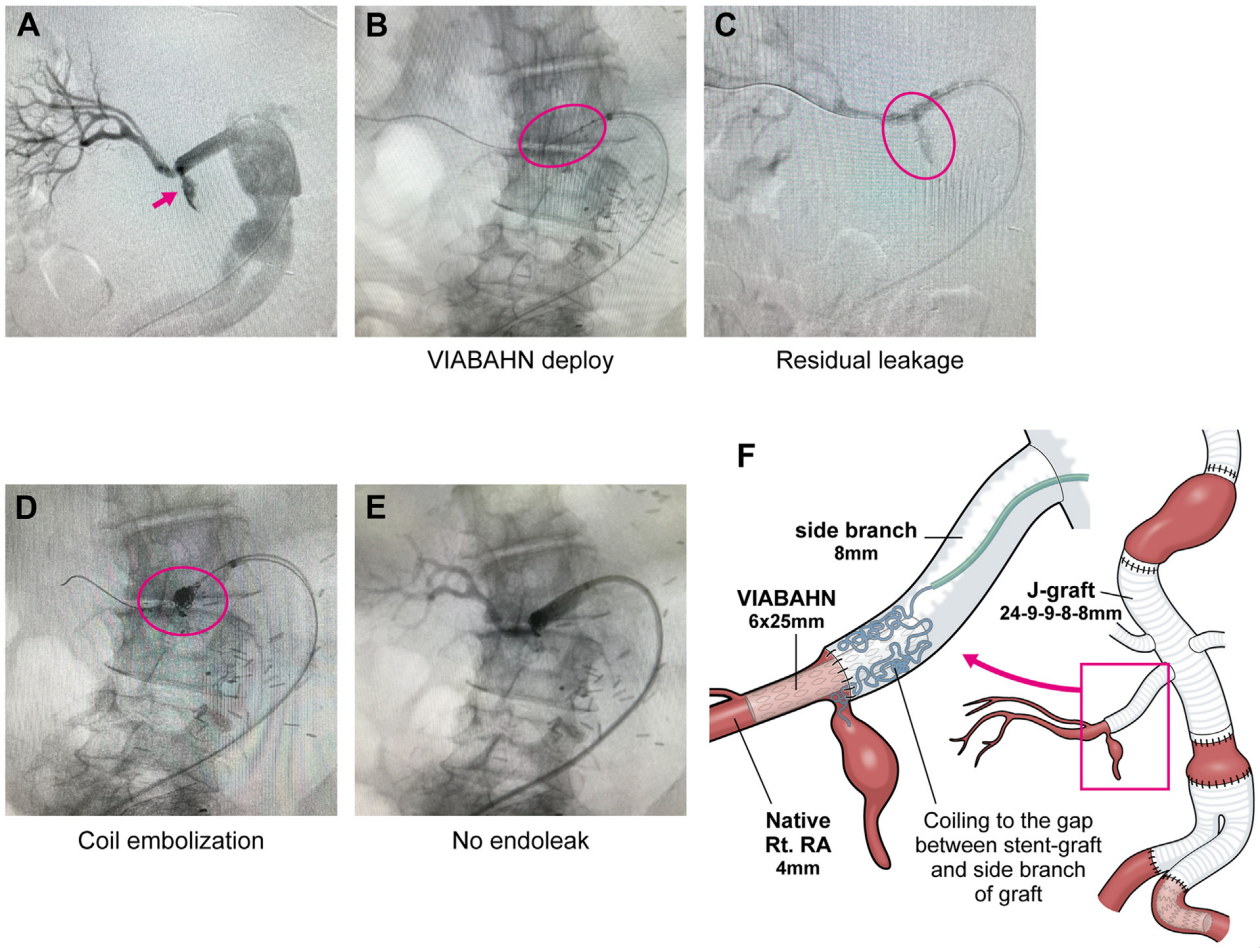
**(A)** Selective right renal artery angiography showed the lesion of anastomotic leakage (*pink arrow*). **(B)** Viabahn stent graft was deployed across the anastomotic leakage site. The viabahn is circumscribed by the *pink circle*. **(C)** The residual endoleak was still detected on the angiography after Viabahn stent graft placement. The *pink circle* delineates the endoleak. **(D)** Coil embolization to fill the gap between Viabahn stent graft and the graft. The *pink circle* outlines the coil embolization. **(E)** The endoleak disappeared after coil embolization. **(F)** The procedural schema was demonstrated.

**Fig 3:**
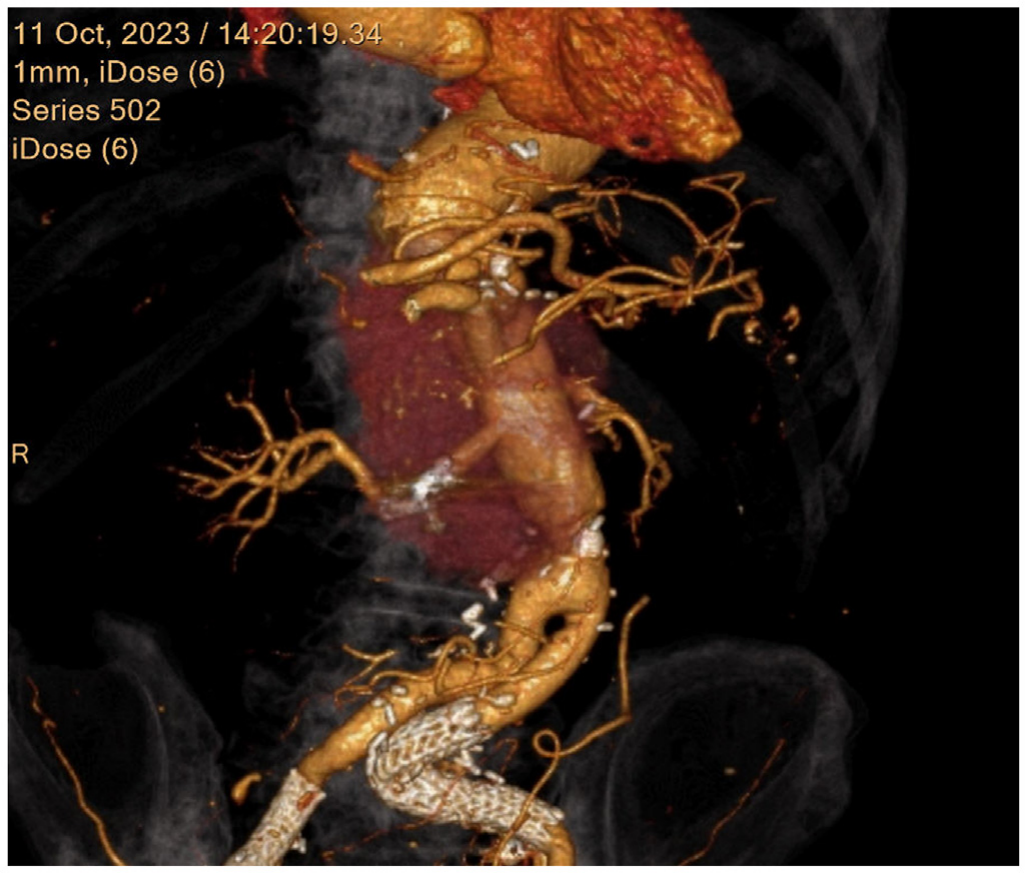
Postoperative contrast-enhanced computed tomography demonstrated no endoleak into the pseudoaneurysm sac; blood flow to the right kidney was maintained.

Under general anesthesia, a 6F guiding sheath was introduced via the right femoral artery and advanced into the side branch of the graft anastomosed to the right renal artery. Digital subtraction angiography confirmed the site of anastomotic rupture (Fig 2, *A*). A 5F Cobra catheter (Terumo, Japan) was then inserted through the 6F guiding sheath and navigated across the anastomotic rupture. Following the exchange of the guidewire for a 0.018-inch extra-stiff guidewire (Cook Medical, Bloomington, IN), a 6 × 25-mm Viabahn stent graft (W. L. Gore & Associates, Flagstaff, AZ) was deployed across the rupture site (Fig 2, *B*). Despite adequate balloon expansion, digital subtraction angiography revealed a type Ia endoleak owing to a diameter mismatch between the stent graft (6 mm) and the side branch of the graft (8 mm) (Fig 2, *C*). To address this issue, a microcatheter was advanced near the anastomotic site through the space between the prosthetic graft and the stent graft, enabling coil embolization using hydrogel-coated coil (Terumo Corporation, Tokyo, Japan) to fill the gap and occlude the ostium of the pseudoaneurysm (Fig 2, *D*). Following additional coil embolization, the pseudoaneurysmal inflow was successfully eliminated (Fig 2, *E*). The procedural schema is illustrated in Fig 2, *F*. Postoperative contrast-enhanced computed tomography confirmed the absence of endoleak at the pseudoaneurysm, with preserved blood flow to the right kidney (Fig 3). The patient was subsequently transferred to a rehabilitation hospital without any deterioration in renal function.

## Discussion

Anastomotic pseudoaneurysm is relatively rare late phase complication after graft replacement^1^ owing to technical failure or infection.^3^ Although open surgical repair is the traditional treatment for anastomotic pseudoaneurysm,^4^ the mortality and morbidity of surgical intervention remains high because of redo procedure and degenerative anastomotic tissue. The high invasiveness of surgical repair would be intolerable for the patients with high frailty. Recently, endovascular repair for pseudoaneurysm has been reported and could be a reasonable and less-invasive alternative to open surgical repair.^5^, ^6^, ^7^ The representative procedures of endovascular repair were stenting and coil embolization. We opted for endovascular repair with Viabahn stent graft, because coil embolization would deteriorate the renal dysfunction and might necessitate dialysis. In our case, the mismatch between the branch graft size and the native renal artery diameter was a concern. After deployment of the stent graft, the gap between the stent graft and the native renal artery diameter and the endoleak from this gap was shown. Preoperatively, we planned two approaches for filling this gap, which were the deployment of another stent graft and coil embolization of the gap. Because we were concerned about the former option in that the additional stent graft would be too oversized for the first deployed stent graft and the misalignment of stent grafts might cause an endoleak into the pseudoaneurysm, coil embolization of the gap was chosen, because it could exclude the pseudoaneurysm completely.

The choice between a self-expanding and balloon-expandable stent graft remains a matter of discussion. From the perspective of accommodating the diameter discrepancy between the surgical graft and the native right renal artery, balloon-expandable stent grafts offer the advantage of allowing for tailored tapering through controlled balloon inflation. However, in cases of anastomotic pseudoaneurysm such as the present case, excessive balloon inflation may exacerbate the fragility of the anastomotic site and potentially lead to rupture during the procedure. In contrast, the self-expanding stent graft used in this case may provide suboptimal attachment to the prosthetic graft, but offers a theoretical advantage in terms of decreased mechanical stress on the anastomotic disruption. Furthermore, because the native renal artery distal to the anastomosis was markedly tortuous, conformability was an important factor, and the use of a self-expanding device was preferred for this anatomical configuration.

Regarding coil embolization for type I endoleak, there is a potential risk of coil migration into the distal renal artery or the pseudoaneurysm sac. However, in this case, adequate oversizing of the Viabahn stent graft relative to the native renal artery was achieved, and no evidence of a type I endoleak was observed, suggesting that the risk of distal migration was minimal. To further mitigate the risk of migration into the pseudoaneurysm sac, hydrogel-coated coils were used. Postoperative contrast-enhanced computed tomography performed in the early phase did not demonstrate any coil migration, although regular imaging follow-up remains warranted.

## Conclusions

Endovascular repair of visceral artery anastomotic pseudoaneurysms after thoracoabdominal aortic repair using stent grafting and coil embolization represents a highly effective treatment strategy, although regular follow-up is necessary to monitor potential migration of the stent graft and coils.

## Funding

None.

## Disclosures

None.
